# Evaluation of Popular Food Trends in K–12 Schools: Nutritional Analysis of Recipes and Daily Serving Patterns

**DOI:** 10.1093/nutrit/nuaf290

**Published:** 2026-05-26

**Authors:** Marjuyua Lartey Gibson, Ruaa Al Juboori, Jane Peterson, Patrick Garmong, Shannon Fitzgerald, Aleshia Hall-Campbell

**Affiliations:** Institute of Child Nutrition (ICN) Applied Research Division, College of Education and Human Sciences, The University of Southern Mississippi, Hattiesburg, MS, 39406, United States; ICN, School of Applied Sciences, The University of Mississippi, Oxford, MS, 38677, United States; Department of Public Health, School of Applied Sciences, The University of Mississippi, Oxford, MS, 38677, United States; Institute of Child Nutrition (ICN) Applied Research Division, College of Education and Human Sciences, The University of Southern Mississippi, Hattiesburg, MS, 39406, United States; ICN, School of Applied Sciences, The University of Mississippi, Oxford, MS, 38677, United States; ICN, School of Applied Sciences, The University of Mississippi, Oxford, MS, 38677, United States; ICN, School of Applied Sciences, The University of Mississippi, Oxford, MS, 38677, United States

**Keywords:** food trends, K-12, school lunch, school breakfast, sodium, sugar, policy, school menus

## Abstract

**Objectives:**

The aims were to (1) identify the frequency of consumption of top food trends in schools participating in the National School Lunch Program (NSLP) and School Breakfast Program (SBP), (2) examine the average nutrient content of recipes for the top menu trends, and (3) quantify the current added-sugar and sodium levels in core recipes to establish baseline data for subsequent modifications.

**Background:**

The US Department of Agriculture (USDA) Food and Nutrition Service has issued a final rule regarding the school meal standards, aligning with the 2020–2025 Dietary Guidelines for Americans. Key updates include a gradual reduction in sodium and new product-based limits on added sugars. Identifying modifiable school menu items is essential to ensuring the NSLP and SBP can meet the needs of the student population.

**Methods:**

This research used a multiphase approach investigating menu trends in K–12 and modifiable school menu options. Popular menu trends were identified using data from a 2024 survey of 1691 school nutrition professionals. A follow-up survey assessed the frequency of consumption of these trends. Recipes for top menu items were sourced from common online repositories, ensuring flexibility for modification to meet nutritional guidelines. Nutritional analysis of selected recipes was conducted using USDA-approved Health-e Pro software (Health-e Pro; Anacortes, WA, USA). Descriptive statistics were used to analyze nutrient content and the frequency of menu trends.

**Results:**

Popular breakfast items, such as muffins, yogurt parfaits, and breakfast smoothies, were major contributors to added-sugar content, while sausage biscuits and breakfast pizza were highest in sodium. Lunch items, including flavored wings and walking tacos, demonstrated high sodium levels, with flavored wings averaging over 2100 mg per serving. Condiments such as BBQ sauce and honey mustard also contributed added sugars and sodium.

**Conclusion:**

While many top food trends are widely served in schools, some exceed recommended thresholds for sodium and added sugars. This study highlights the potential to modify existing recipes using herbs, spices, and ingredient substitutions to meet the USDA’s updated school meal standards by 2027, without compromising student acceptability.

## INTRODUCTION

The US Department of Agriculture (USDA) Food and Nutrition Services (FNS) is responsible for the administration of Child Nutrition Programs at the federal level, such as the National School Lunch Program (NSLP), School Breakfast Program (SBP), Fresh Fruit and Vegetable Program, and the Special Milk Program.^1^^,^^2^ Although the impact of these programs varies depending on the population they serve, the NSLP and the SBP combined provided 7 048 690 000 meals to school-aged children in fiscal year 2023.^3^ Because school meals can contribute up to one-half of the child’s daily calorie needs, it is essential to consider the nutrient makeup of the food served and consumed in the NSLP and SBP.^4^

In 2010, the Healthy, Hunger-Free Kids Act (HHFKA) transformed school meal standards by increasing access to fruits, vegetables, and whole grains, while simultaneously decreasing saturated fats, eliminating *trans* fats, and imposing limits on sodium by grade level.^5^ Multiple research studies have documented the impact of the 2010 HHFKA on school meal participation and meal quality.^6–11^ In 3 studies^7–9^ that examined the impact of the 2010 HHFKA on school NSLP meal participation, overall, there were no statistically significant changes in participation between baseline and the years that followed the implementation. Interestingly, 2 of the studies found that participation rates among students eligible for reduced-price lunches were significantly higher 1 year after the implementation of the HHFKA versus the year of implementation or at baseline for the studies’ data collection.^7^^,^^8^ In previous research assessing the impact of the HHFKA on meal quality, the overarching theme has been that meals served after the implementation of the HHFKA have resulted in increased selection and consumption of fruits and vegetables^9^^,^^10^ and in a positive impact on micro- and macronutrient intake of calories from total fat and saturated fat, carbohydrates, dietary fiber, cholesterol, sodium, and vitamin C.^11^

The School Nutrition and Meal Cost (SNMC) Study examined the nutritional quality of the NSLP and SBP using the Healthy Eating Index-2010 (HEI-2010).^6^ The researchers found that the nutritional quality of the school breakfast meals had a mean score of 71.3 out of a possible 100 and school lunches had a mean total HEI-2010 score of 81.5 out of a possible 100, suggesting that the 2010 HHFKA had a substantial impact on the nutritional quality of breakfast and lunches served to children participating in the SBP and NSLP.^6^ These numbers show a statistically significant increase in the quality of school meals as a result of the updated standards. The SNMC study also examined the nutrition standards for SBP and NSLP meals associated with the HHFKA, including sodium limits. Target 1 sodium levels range from 540 mg or less to 640 mg or less for breakfast, and target 1A sodium levels range from 1110 mg or less to 1280 mg or less for lunch per week, depending on the age/grade of the students served.^5^ At the time of the study, approximately 72% of average weekly lunch menus and 67% of average weekly breakfast menus met the target 1 sodium school meal requirements.^6^

While school meal pattern regulations have significantly improved the meals served, some areas of improvement are still needed. Researchers using the data from the SNMC study found that, on average, 92% of school breakfast meals and 69% of school lunches exceeded the Dietary Guidelines for Americans (DGA) limit for added sugar (no more than 10% of calories from added sugar daily).^12^ The researchers found that the main contributor of added sugar in school meals was flavored skim milk, followed by sweetened cold cereals and condiments.^12^ Chapman et al^13^ examined the daily saturated fat and sodium content of elementary school meals across 128 school districts for all plausible daily breakfast and lunch reimbursement meal combinations. The study found that there can be significant variations in the sodium content of meals served at breakfast and lunch in schools participating in the study. However, on average, 11% of all meal combinations for breakfast had more than 540 mg of sodium and 12.4% of all lunch meal combinations had more than 1230 mg of sodium. Additionally, the study showed that the leading sources of sodium for lunch entrées were deli entrées (942 mg of sodium), hot sandwiches (766 mg of sodium), and entrée salads (744 mg of sodium), and breakfast entrées with the greatest amount of sodium were breakfast sandwiches.^13^ In late April 2024, the USDA FNS published a final rule based on the *Dietary Guidelines for Americans, 2020*–*2025*, that includes some updates to the 2010 HHKFA.^14^ Notable changes include new requirements regarding added sugars in school meals. The final rule requires that no more than 10% of weekly calories in school meals come from added sugars and will require product-based added-sugar limits for breakfast cereals (no more than 6 g of added sugar per ounce), yogurt (no more than 12 g of added sugar per 6 ounces), and flavored milk (no more than 10 g of added sugar per 8 fluid ounces) by Fall 2027. The final rule also addresses sodium in school meals, denoting a modification to the current meal pattern standard.^14^ By Fall 2027, the final rule requires that breakfast and lunch reach a one-time reduction in current sodium levels of 10% for breakfast and 15% for lunch.^14^

According to the USDA Agricultural Research Service, approximately one-third of American children ages 2 to 19 years receive 14% of their total energy from added sugars, which is higher than the current recommendation from the DGA. The study, which used What We Eat in America, National Health and Nutrition Examination Survey (NHANES) 2015–2016 data and the Food Patterns Equivalents data 2015–2016, identified major food and beverage sources of sugars in American diets. The main sources of added sugars for school-aged children identified in the study are sweetened beverages, sweet bakery products, and other desserts, with soft drinks, fruit drinks, and sports and energy drinks being the top contributors.^15^ Foods and beverages, such as ready-to-eat cereals and flavored milk, which are served in school meals, were indicated as sources of added sugar for children ages 6–11 years and 12–19 years^15^ and indicated as 1 of the top 3 consumed breakfast foods and beverages for children and teens ages 2–19 years in another study using 2015– 2018 NHANES data.^16^ Considering these findings and the idea that children consume up to two-thirds of their nutrients in the school setting, efforts made by the USDA FNS to align sugar intake with that of the DGA may be a critical step in improving the diet quality of school-aged children.

The 2020–2025 DGA recommend that persons older than 14 years of age consume less than 2300 mg of sodium per day, and those younger than that should consume even less sodium.^17^ Several studies have examined the prevalence of sodium intake among Americans using NHANES data from various data-collection periods.^16^^,^^18^^,^^19^ In the NHANES 2009–2012 data, over 92%–94% of children ages 2–18 years participating in the research had sodium intakes that exceeded the 2015–2025 DGA recommendations, with the top food sources being bread, deli meats, pizza, poultry, sandwiches, soups, cheese, pasta dishes, mixed meat dishes, and savory snacks.^18^ Another study analyzing 2003–2016 NHANES data indicated that the median sodium intake was 2507 mg per day for ages 4–8 years, 2934 mg per day for ages 9–13 years, and 3124 mg per day for ages 14–17 years.^19^ These findings indicate that sodium intake in the school-aged population is persistently elevated, and there is some importance in identifying ways to mitigate excessive sodium intake in the pediatric population.

Controlled studies in adult populations have investigated the use of herbs and spices as a strategy to maintain food consumption and palatability when reducing ingredients such as sodium, sugar, or fat.^20–22^ Petersen and colleagues^22^ investigated the reformulation of 10 commonly used foods, identified from the NHANES data from 2015–2018, to be lower in fat, sodium, and added sugars using herbs and spices as flavor enhancers. Through blind taste testing with consumers, the researchers found that the overall liking ratings of 7 of the 10 reformulated foods were equal to or superior to the original version. However, 3 of the 10 reformulated dishes were not as well received. Cheese pizza, macaroni and cheese, and chicken pot pie were most acceptable in the original version.^22^ Research on the influence of herbs and spices on the palatability of reduced-fat foods demonstrated that adding herbs and spices to a reduced-fat version of certain foods could restore or significantly improve overall liking of those foods, often bringing the liking level close to that of their full-fat counterparts.^20^ Similarly, researchers conducted 2 controlled studies examining the influence of spices on the overall consumer liking of 3 different recipe varieties (full sugar, reduced sugar, and reduced sugar with added spice) for tea, oatmeal, and apple crisps.^21^ Based on the research findings, adding spices did not consistently improve the acceptance of all 3 foods. For example, decreasing the amount of sugar in tea significantly reduced the preference for tea, and adding spices did not have an impact on the mean liking of tea. As for oatmeal, reduced sugar and reduced sugar with spice were less liked than full sugar oatmeal. However, the hedonic pleasure and overall liking of an apple crisp with reduced-sugar and spices were significantly maintained, making the reduced-sugar-with-spices version comparable to the full-sugar version. This maintenance of palatability with reduced sugar was associated with no significant difference in the amount of apple crisp consumed between the full-sugar, reduced-sugar, and reduced-sugar-with-spice versions in the study. ^21^ These findings suggest that incorporating herbs and spices in controlled food settings can maintain palatability, which can be a strategy to support healthier dietary choices and potentially influence the intake of less-healthy components such as sodium, fat, or sugar.

Given the changes in the final rule for added sugars (<10% of calories from added sugar across the school week for lunch and breakfast) and sodium (target 1 for breakfast, ≤540 mg to ≤640 mg per school week [depending on grade level], and target 1A, ≤1110 mg to ≤1280 mg per school week [depending on grade level])^5^and the potential impact of reformulated popular food trends in K–12 settings, identifying modifiable school menu items is essential. This study (1) identifies the frequency of consumption of top food trends in schools participating in the NSLP and SBP, (2) analyzes the nutritional content of their recipes, and (3) quantifies the current added-sugar and sodium levels in core recipes to establish baseline data for subsequent modifications.

## METHODS

This research used a multiphase approach to investigate food trends in K–12 schools participating in the NSLP and SBP. Four primary questions guided the study:

1. What are the most popular breakfast and lunch food trends in K–12 schools participating in the NSLP and SBP?
2. Among the identified trends, how frequently are these food items served?
3. To what extent are standardized recipes for the top food trends available through online resources and culinary databases used by school food service operators?
4. What is the nutritional profile of representative recipes for the top food trends, as analyzed by Health-e-Pro software (Health-e-Pro, Anacortes, WA, USA), and how do these profiles compare to USDA school meal pattern guidelines for sodium and sugars?

### Identifying Popular Food Trends

In April 2024, the Institute of Child Nutrition (ICN) Applied Research Division recruited school nutrition professionals to participate in a Food Trends in K–12 study (unpublished data). In total, 1691 school nutrition professionals participated in an online survey detailing food items currently popular, increasing in popularity, or decreasing in popularity across K–12 schools. The survey specifically addressed breakfast and lunch items served under the NSLP and SBP. Details of the 2024 Food Trends in K–12 study have been published in a separate article.^24^

### Frequency of Consumption

Based on the findings from the 2024 Food Trends in K–12 study, the researchers created a separate survey, the Top Food Trends in K–12: Frequency of Consumption survey, to identify the frequency with which the popular food items identified in the first study were served in schools. The frequency survey was conducted in November and December 2024. Of the 2665 individuals who consented, 1594 (59.8%) did not proceed beyond the first question and were excluded, which yielded a final analytic sample of 1071 school nutrition professionals (directors, managers, supervisors, and frontline staff). Recruitment was conducted through the ICN website, social media channels, and direct mail invitations. Eligibility required knowledge of local school meal consumption. The frequency of consumption survey allowed the researchers to confirm the top modifiable food items served in school districts nationwide. The survey was approved by the Institutional Review Board at the University of Southern Mississippi. The survey captured respondent role, USDA FNS region, district enrollment category, and educational setting. Our survey did not collect socioeconomic measures or urbanicity; therefore, we could not stratify recipe availability by socioeconomic status or rural–urban context.

### Standardized Recipes

Researchers conducted searches across multiple commonly used recipe repositories within the school nutrition community, including the ICN Child Nutrition Recipe Box, Healthy School Recipes, Conagra Food Service, and others, to identify recipes for the top foods selected from the Frequency of Consumption survey. Researchers identified a maximum of 3 recipe variations for each of the top food items, based on food trends data. Recipes were considered acceptable if they met the general definition of the food item and allowed for flexibility in modification, including ingredient substitutions to meet dietary needs, cooking method adjustments, and the potential to reduce sodium or sugar. Items were excluded if they lacked accessible recipes, were not modifiable, or did not have at least 2 available recipe variations. As such, all included recipes were complete; no imputation or exclusions for implausible values were required.

### Nutrient Analysis

The recipes identified for each food item were entered into Health-e-Pro software for nutrient analysis. Health-e Pro is a cloud-based, USDA-approved software platform designed for menu planning and nutrient analysis in compliance with school meal guidelines. The version used in this study (Meal Planning 2024.9) includes monthly updates to ensure alignment with the latest regulatory standards.

### Statistical Analysis

Descriptive statistics, including means, medians, modes, ranges, and SDs, were used to calculate the average nutrient content (calories, sodium, and sugar) of selected breakfast and lunch items. Additionally, the percentages of how often foods in the breakfast, lunch, and sauces/condiments categories were served were calculated. All statistical analyses were performed using SPSS software (IBM Corporation, Armonk, NY, USA).

## RESULTS

A total of 1071 individuals responded to the Top Food Trends in K–12: Frequency of Consumption survey. Among respondents, 46.8% were school nutrition managers/supervisors and 40.0% were school nutrition directors. The largest share of participants was from the Midwest USDA FNS region (20.4%), followed by the Southwest (19.6%) and Southeast (15.5%). Most respondents served smaller districts (≤2500 students; 55.0%), and 42.2% reported working with all schools (K–12). **Table 1** provides the demographic characteristics of the respondents to the Top Food Trends in K–12: Frequency of Consumption survey.

Service patterns (**Table 2**) show that muffins are common (36.5% at least weekly; 17.4% never), yogurt parfaits are less routine (24.6% at least weekly; 38.3% never), and smoothies are infrequent (11.5% at least weekly; 74.2% never). Lunch pizza is widely offered (47.0% at least weekly; 3.5% never), chicken tenders are regular (30.0% at least weekly; 2.8% never), while flavored wings are rare (5.4% at least weekly; 66.4% never), and walking tacos are occasionally offered (5.5% at least weekly; 44.9% never).

**Table 1.** Demographic Characteristics of the Respondents

Characteristics	No.	%
Job title		
School Nutrition Manager/Supervisor	501	46.8
School Nutrition Director	428	40
School Nutrition Menu Planner	78	7.3
School Nutrition Chef	62	5.8
Missing	2	0.2
Total	1071	100
Region		
Mid-Atlantic	75	7
Midwest	219	20.4
Mountain Plains	89	8.3
Northeast	79	7.4
Southeast	166	15.5
Southwest	210	19.6
Western	103	9.6
Missing	130	12.1
Total	1071	100
Enrollment		
<1000	427	39.9
1000–2500	162	15.1
2501–9999	188	17.6
10 000–29 999	94	8.8
>30 000	70	6.5
Missing	130	12.1
Total	1071	100
Setting		
Elementary school (grades K–5)	348	32.5
Middle school (grades 6–8)	213	19.9
High school (grades 9–12)	127	11.9
All schools (grades K–12)	452	42.2
Missing	130	12.1
Total	1071	100

*n* = 1071.

**Table 2.** Frequency of Service for Popular K–12 Menu Items Over a 4-Week Period

Item	At least weekly, *n* (%)	Monthly, *n* (%)	Never, *n* (%)
Breakfast items			
Breakfast pizza	206 (19.3)	478 (44.7)	385 (36.0)
Breakfast smoothie	124 (11.6)	150 (14.0)	795 (74.3)
Donuts	214 (20.0)	373 (34.9)	481 (45.0)
Muffins	391 (36.5)	492 (46.0)	186 (17.4)
Sausage biscuit	224 (20.9)	386 (36.1)	459 (42.9)
Lunch items			
Hamburger	278 (28.4)	662 (67.6)	40 (4.1)
Pizza	502 (51.2)	441 (45.0)	37 (3.8)
Chicken tenders/nuggets	322 (32.9)	628 (64.1)	30 (3.1)
Nachos	137 (14.0)	603 (61.5)	240 (24.5)
Flavored wings	58 (5.9)	212 (21.6)	710 (72.4)
Walking tacos	59 (6.0)	441 (45.0)	480 (49.0)
Sauces and condiments			
Ranch dressing	842 (86.1)	99 (10.1)	37 (3.8)
Hot sauce	378 (38.7)	176 (18.0)	424 (43.4)
Honey mustard sauce	265 (27.1)	214 (21.9)	499 (51.0)
BBQ sauce	435 (44.5)	420 (42.9)	123 (12.6)

Missing data: breakfast items had 2 missing responses (*n* = 1069); lunch items had 91 missing responses (*n* = 980 for each lunch item); sauces/condiments had 93 missing responses (*n* = 978 for each). Percentages derived from item-specific valid *n* values.

**Table 3** presents descriptive statistics for calories, sodium, and added sugar by recipe category for breakfast, lunch, and condiments identified as popular foods served in school nutrition programs. Across breakfast and lunch menu items (**Table 3**), added sugars were highest for yogurt parfaits (18.67 ± 18.28 g) and breakfast smoothies (15.02 ± 0.00 g), followed by muffins (5.21 ± 6.56 g) and flavored wings (5.55 ± 7.84 g), while sausage biscuits and breakfast pizza were low in added sugars (2.00 ± 1.00 g and 0.22 ± 0.39 g).

**Table 3.** Nutrient Profiles of Representative Recipes for Popular K–12 Menu Items

Item	Calories, kcal	Sodium, mg	Added sugars, g
Breakfast			
Sausage biscuit	388.18 ± 199.31 (160.00-528.20)	593.41 ± 219.76 (340.00-731.60)	2.00 ± 1.00 (1.00-3.00)
Breakfast pizza	361.21 ± 113.93 (234.18-454.34)	833.17 ± 384.41 (600.37-1276.80)	0.22 ± 0.39 (0.00-0.67)
Muffin	174.26 ± 6.15 (170.55-181.36)	233.97 ± 118.44 (161.24-370.64)	5.21 ± 6.56 (0.00-12.57)
Yogurt parfait	386.90 ± 170.50 (191.25-503.68)	208.77 ± 78.03 (118.97-260.07)	18.67 ± 18.28 (3.84-39.09)
Breakfast smoothie	149.74 ± 75.21 (64.59-207.08)	46.45 ± 36.83 (3.97-69.32)	15.02 ± 0.00 (15.02-15.02)
Lunch			
Pizza	217.70 ± 106.77 (99.48-307.12)	368.37 ± 69.31 (299.39-438.01)	NA
Chicken tenders	161.62 ± 8.91 (151.33-167.08)	334.83 ± 81.16 (241.25-386.01)	0.06 ± 0.08 (0.00-0.15)
Nachos	372.84 ± 169.59 (238.51-563.41)	514.75 ± 69.41 (458.34-592.26)	NA
Flavored wings	883.92 ± 240.27 (651.66-1131.40)	2131.60 ± 376.41 (1747.30-2499.60)	5.55 ± 7.84 (0.00-11.09)
Walking tacos	516.30 ± 170.76 (324.91-653.05)	781.35 ± 158.17 (599.01-881.44)	1.80 ± 2.30 (0.00-4.39)
Condiments			
Ranch dressing	74.06 ± 36.13 (34.25-104.76)	141.83 ± 64.65 (67.60-185.81)	NA
Honey mustard sauce	136.28 ± 12.00 (127.80-144.77)	145.97 ± 4.05 (143.10-148.83)	NA
BBQ sauce	41.77 ± 26.82 (25.24-72.72)	211.60 ± 185.80 (89.08-425.39)	6.16 ± 6.16 (1.81-10.51)

Values are mean ± SD (min-max) for calories, sodium, and added sugars.

Abbreviations: max, maximum; min, minimum; NA, not applicable or not reported for added sugars in that item.

When examining the sodium levels in breakfast and lunch menu items, sodium amounts were greatest for flavored wings (2131.60 ± 376.41 mg), with walking tacos (781.35 ± 158.17 mg) and nachos (514.75 ± 69.41 mg) also elevated in sodium. Breakfast pizza (833.17 ± 384.41 mg) and sausage biscuits (593.41 ± 219.76 mg) were the higher-sodium breakfast items.

**Table 3** also presents the average added-sugar amounts and sodium values for condiments frequently served in school meals. The only condiment with a represented amount of added sugars was honey mustard (6.16 ± 6.16 g). The condiment with the highest sodium levels was barbeque sauce (211.16 ± 185.60 mg), followed by honey mustard sauce (145.97 ± 4.05 mg) and ranch dressing (141.83 ± 64.65 mg).

## DISCUSSION

This research provides valuable insights for school nutrition professionals seeking to balance nutritional requirements, student preferences, and operational constraints by identifying top food trends with modifiable recipes to meet the School Nutrition Standards Final Rule^14^ for sodium reductions and added-sugar limits. In analyzing top food trends for school nutrition programs (SNPs), a careful and deliberate selection methodology was used to ensure the relevance and applicability of the items studied. The selection criteria focused primarily on the availability of multiple recipes or preparation methods, the potential for modification of ingredients or preparation techniques, alignment with current food trends in school nutrition, compatibility with USDA meal guidelines, and feasibility of preparation within typical school food service facilities.

Despite their popularity in trend reports, certain menu items were excluded from the analysis based on specific factors. Items with limited recipe availability (1 or no recipes found in common school nutrition recipe databases), those that are predominantly heat-and-serve with minimal opportunity for ingredient or preparation modifications, and items with a high likelihood of being sourced as prepackaged in most SNPs were omitted from the study.

Based on our analysis using Health-e Pro software, the greatest contributors to added sugar for school breakfast and lunch varied based on the recipes available in the recipe repository. Average levels of added sugar in school breakfast menu items ranged from less than 1 g to approximately 15 g of added sugar, with yogurt parfaits (∼18.7 g of added sugar), breakfast smoothies (∼15 g of added sugar), and muffins (∼7.8 g of added sugar) ranked highest. These findings are similar to those found in previous studies. Fox and colleagues^6^ found that the top contributors to added sugar in school breakfasts included toaster pastries, breakfast bars, muffins, sweet/quick breads, and low-fat or fat-free yogurt.

Average levels of added sugar in school lunches and condiments also varied but were lower than that of most breakfast menu items, with an average range of zero to nearly 4 g of added sugars for top lunch menu items in this study. Flavored wings (range of 0–11.1 g of added sugar) and tacos (range of 0–4.39 g of added sugar) were the top-ranked sodium contributors for lunch, and barbeque sauce (range of 1.8–10.5 g of added sugar) was the top-ranked added-sugar contributor for condiments and sauces. These lunch and condiment findings were similar to previous research as well.^6^^,^^12^ Fox and colleagues^12^ identified condiments as a top source of added sugar in school lunches in their study that assessed the levels of added sugar in school meals. While the new school meal standards for added sugar are initially directed towards product-based limits (breakfast cereal, yogurt, and flavored milk) and extend to weekly limits in the school year 2027–2028, there is an opportunity for school nutrition professionals to explore recipe development to meet the challenges of serving students’ desired menu items while meeting the USDA school meal standards for added-sugar and calorie limits. The popular breakfast items identified in this study could benefit from recipe modifications that lower the use of added sugars and substitute added sugars for alternative spices, such as cinnamon, ginger, cardamon, nutmeg, and allspice, as demonstrated in the study by Petersen et al.^22^ Modifications like these may have little to no negative impact on student consumption, but the modifications must include student testing for acceptability. Additionally, school food service directors and staff may benefit from additional assistance as they implement and maintain school meal nutrition standards for breakfast and lunch, including menu development, increased financial support, culinary training, and creative student engagement.

The leading contributors to sodium levels for breakfast items in the current study were sausage biscuits and breakfast pizzas. The average sodium level for sausage biscuit recipes identified in recipe repositories for school nutrition professionals was 593 mg, which exceeds the daily breakfast sodium limits for grades K–5 under the current USDA nutrition standards.^1^ The breakfast pizza’s average sodium level was 833 mg, based on recipes identified in this study. This amount of sodium exceeds the current daily breakfast sodium limits for all age groups. While each of the lunch menu items analyzed in this study had higher sodium levels than breakfast items, not all of the lunch menu items exceeded the current daily sodium limits for either age or grade group. For example, the average sodium levels for nachos (514 mg of sodium) meet nearly half the daily sodium limit for grades K–5, and the average sodium levels for walking tacos (781 mg of sodium) were greater than half the current daily sodium limits for all age/grade groups. However, the average sodium levels for flavored wings (2131 mg) nearly doubled the sodium limit in each age/grade category based on the current nutrition standards for school meals.

These findings are consistent with prior research on the sodium content of school meals. For example, a study by Chapman and colleagues ^13^ in 128 school districts found that breakfast entrées with the highest sodium content were those that included meat and cheese, such as sausage biscuits, and the lunch entrées for highest sodium content were deli entrées, hot sandwiches, entrée salads, Mexican-style foods, hot dogs, and breaded chicken. These results are consistent with our findings, highlighting the hidden sodium in popular menu items among school-aged children. Additionally, results from the current study complement prior research showing the top food sources for sodium in the American diet.^16^^,^^18^^,^^23^ In a study conducted by Ahmed and colleagues,^23^ the top food categories that accounted for greater than 51% to 56.3% of the total dietary sodium intake for children ages 4 to 18 years included pizza; breads, rolls, and buns; cold cuts/cured meats; soups; burritos and tacos; poultry; cheese; bacon, hot dogs, and sausages; and chicken nuggets.^23^ Similar findings were noted in the NHANES data mentioned previously.^18^

While the USDA school meal standard for sodium is based on the sodium found in school meals offered on an average week at local schools, it is important to look at how much sodium individual food items contribute to the weekly allowance. The top sodium contributors in school lunch are likely students’ favorite menu items, both within and outside of school hours. As indicated in previous research on the subject, many Americans consume sodium at higher-than-warranted levels.^12^^,^^16^^,^^18^^,^^19^ The challenge for the school nutrition professional is to create menu items that appeal to the student population but meet the nutritional standards established by the USDA FNS. Different strategies can be used to tackle the higher levels of sodium in popular menu items in K–12, including the development of scratch or semi-scratch recipes that offer alternatives to salt that provide flavor profiles that are suitable for the student palate, or serving more fresh fruits and vegetables (in salad bars) and carefully reading food labels. School nutrition professionals also have the responsibility of ensuring that a balance of sodium is offered over the course of the week, which requires strategic meal planning.

## CONCLUSION

In this project, the selection of top food items carefully balanced the need for trendy, appealing options with the practical realities of SNPs. By focusing on items with multiple recipe options and significant modification potential, the study offers valuable insights that can be readily implemented in diverse school food service environments. While excluding certain trending items may seem limiting in scope, it allows for a more targeted and applicable analysis of foods that can be meaningfully adapted to meet nutritional standards, flavor enhancements, and student preferences within the unique constraints of SNPs.

In this project, the researchers analyzed, on average, 3 recipes for each popular food item, providing a more applicable look at added-sugar and sodium content in the selected foods. The results of this study can be used to justify the modification of commonly accessed recipes using herbs and spices to create an acceptable flavor profile for school-aged children and likely provide a meaningful reduction in the amount of added sugars and sodium consumed in school meals.

The present research contributes important information pertaining to the potential reformulation of recipes and may have a significant impact on school nutrition recipe development. Future analyses examining the sodium and sugar contributions of other foods and food categories in school meals could provide additional insight into recipe development and possibly product reformulation efforts. Although the food used in this study was informed by a food trends study, there are still some possible differences in sodium and sugar levels across different food groups and food categories, which may have gone unidentified based on the responses of those who chose to participate in the initial research. Further research should aim to examine sodium contributors in school meals and across additional demographic variables.

## Data Availability

The data that supported the findings of this study were collected by the authors. Due to ethical considerations, the data are not publicly available. The identified data may be shared upon request, subject to IRB approval.
